# Efficacy and safety of SDT‐001, a dual‐task digital device, in managing attention‐deficit/hyperactivity disorder symptoms in children and adolescents: a phase 3, randomized, standard treatment‐controlled study

**DOI:** 10.1111/pcn.13833

**Published:** 2025-05-02

**Authors:** Katsunaka Mikami, Tasuku Miyajima, Ryo Nishino, Naohiro Kawazoe, Yousuke Kinoshita, Takashi Okada, Hiroki Fukuju

**Affiliations:** ^1^ Department of Psychiatry Tokai University School of Medicine Kanagawa Japan; ^2^ Tokyo Kasei University Tokyo Japan; ^3^ Institute for Advancement of Clinical and Translational Science (iACT) Kyoto University Hospital Kyoto Japan; ^4^ Drug Development and Regulatory Science Division, Shionogi & Co., Ltd. Osaka Japan; ^5^ Department of Psychiatry Nara Medical University Nara Japan

**Keywords:** attention‐deficit/hyperactivity disorder, children, cognitive function, digital device, Japan

## Abstract

**Aim:**

This phase 3, multicenter, open‐label study aimed to evaluate the efficacy and safety of SDT‐001, a dual‐task digital device, compared to standard treatment (environmental and/or psychosocial treatment: treatment as usual; TAU) in the comparison part and to evaluate the safety, tolerability, and long‐term efficacy of SDT‐001 in the repetition part in Japanese children and adolescents with attention‐deficit/hyperactivity disorder (ADHD).

**Methods:**

In the comparison part, participants on standard treatment were randomized (2:1) to SDT‐001 (*n* = 109; 25 min/day for 6 weeks, with a 4‐week follow‐up) or TAU (*n* = 55) groups. Participants (*n* = 126) from the comparison part transitioned to a single‐arm repetition part with SDT‐001 (6 weeks and followed for 12 weeks). Primary endpoint in the comparison part was changed from baseline to 6 weeks in ADHD rating scale IV (ADHD‐RS‐IV) inattention scores.

**Results:**

In the comparison part, SDT‐001 demonstrated superiority to TAU, with significantly greater improvements from baseline to week 6 in ADHD‐RS‐IV inattention (adjusted mean difference [95% confidence interval], −2.97 [−4.38, −1.56]; *P* < 0.0001), total (−4.56 [−6.75, −2.38]; *P* < 0.0001), and hyperactivity‐impulsivity (−1.55 [−2.64, −0.46]; *P* = 0.0056) scores. Additionally, other secondary endpoints showed improvements in symptoms in the SDT‐001 group. In the repetition part, SDT‐001 showed sustained reductions in ADHD‐RS‐IV scores till 12 weeks after completion of the 6‐week treatment. No new severe adverse events or safety concerns were reported.

**Conclusion:**

SDT‐001 demonstrated superior efficacy at week 6 in ADHD‐RS‐IV compared to TAU, and reductions in scores were maintained up to the following 12 weeks, indicating its potential as a novel digital therapeutic option for ADHD management.

Attention‐deficit/hyperactivity disorder (ADHD) is a common pediatric neurodevelopmental disorder, with hyperactivity‐impulsivity and/or inattention symptoms often persisting into adulthood.^1^ Globally, ADHD prevalence was 7.6% and 5.6% in populations aged 3–12 and 12–18 years, respectively.^1^ In Japan, 838,265 individuals had ADHD diagnosis in fiscal years 2010–2019, with 14.5% aged 0–6, 45.5% aged 7–19, and 40.0% aged >19 years.^2^ Untreated ADHD negatively affects quality of life, impacting physical and psychosocial (academic, social, and emotional) health.^3^, ^4^, ^5^, ^6^

Japanese clinical guidelines recommend school environment management and psychosocial treatment as first‐line, and pharmacotherapy/combination as second‐line ADHD treatment.^7^, ^8^ Pharmacological treatments, like stimulant medications, have well‐documented side effects and may not optimize certain cognitive domains.^9^, ^10^ ADHD treatment adherence is generally low, with decrease and inconsistent medication use after age 13.^11^, ^12^ Nonpharmacological interventions, like cognitive behavioral therapy, present challenges like limited qualified provider availability, clinician variability, and suboptimal patient engagement owing to side effects and addiction risk.^13^, ^14^, ^15^, ^16^ These limitations highlight the need for scalable interventions targeting ADHD‐related attention and cognitive deficits.^17^

A novel digital therapeutic software application, AKL‐T01 has been developed for clinical trials on pediatric ADHD patients.^18^ Previous studies showed significant (*P* < 0.0001) improvement in Test of Variables of Attention (TOVA)‐Attention Performance Index (API) with AKL‐T01 without stimulant in children aged 8–12^19^ and significant improvements in ADHD‐related impairment and symptoms with AKL‐T01 as an add‐on to psychostimulant in children aged 8–14 over two 4‐week treatment periods.^20^ AKL‐T01 demonstrated favorable safety, with no serious adverse device effects and fewer mild–moderate adverse device events (ADEs).^19^, ^20^ EndeavorRx® is the United States‐marketed application that improves AKL‐T01 specifications contributing to entertainment value and security beyond its core mechanisms of dual‐task execution and dynamic adjustment of task difficulty. It is indicated for improving attention function, as measured by computer‐based testing in children aged 8–17 with primarily inattentive or combined‐type ADHD.^18^

Digital therapeutics have improved attention, working memory, and inhibition in children with ADHD,^21^ and multitasking in healthy older adults.^22^ Neurofeedback and cognitive behavioral therapies have been shown to reduce cognitive symptoms of ADHD.^23^ This suggests that digital therapeutics can be effective for improving ADHD symptoms in children and adolescents.^24^ SDT‐001 is an application that translates the language and expressions used in EndeavorRx® into Japanese. Given the rising demand for ADHD treatment, the centrality of cognitive deficits, and the need for expanded access to evidence‐based interventions, we believe that SDT‐001 might provide similar benefits for Japanese children and adolescents with ADHD.

A previous phase 2 study evaluated the efficacy and safety of SDT‐001 compared to a sham control (single‐task device) in Japanese children and adolescents with ADHD. Both groups showed improvements in physician‐rated ADHD rating scale (ADHD‐RS‐IV) total score (ADHD‐RS‐T), and inattention (ADHD‐RS‐I) and hyperactivity‐impulsivity subscale scores (ADHD‐RS‐H) over time, with greater, though not statistically significant, trends of improvement with SDT‐001 compared to single‐task at week 6.^25^ Previous reports suggested that performing a single or dual task activates the prefrontal cortex,^22^, ^26^ thought to be involved in ADHD symptoms. Consequently, the single‐task application was deemed inappropriate as a sham control that must guarantee blinding and have no therapeutic effect. The *post hoc* analysis showed significant improvement in ADHD‐RS‐T, ADHD‐RS‐I, and ADHD‐RS‐H in both SDT‐001 and single‐task groups compared to a non‐randomized observation group (psychosocial treatment including environment adjustment). The improvement was greater with SDT‐001, indicating advantages of SDT‐001 over psychosocial treatment and environmental adjustment in treating ADHD symptoms. Reductions in scores were maintained up to 4 weeks after the end of treatment in both SDT‐001 and single‐task groups. The phase 2 study findings supported primary endpoint selection, effect size estimation for SDT‐001, and sample size calculation in the phase 3 study.

Hence, in this randomized, open‐label, phase 3 study, standard treatment of environmental adjustment and psychosocial treatment (treatment as usual; TAU) was set as the control group instead of sham treatment in the comparison part to evaluate the efficacy and safety of adding SDT‐001 to TAU compared to TAU alone over 6 weeks in Japanese children and adolescents with ADHD. Following the comparison part, an open‐label, single‐arm repetition part aimed to evaluate the safety, tolerability, and sustained efficacy of re‐administering SDT‐001.

## Methods

### Study design

This multicenter, open‐label study (jRCT2042220012; study period: 12 May 2022 to 25 Dec 2023), consisted of comparison and repetition parts (Supplementary Fig. S1). In the randomized, TAU‐controlled, parallel‐group comparison part, eligible participants on standard treatment were randomized (2:1) to SDT‐001 or TAU (see Randomization and blinding section). Participants in the SDT‐001 group received SDT‐001 once daily (approximately 25 min) for 6 weeks, while participants in the TAU group received standard treatment. During the study, standard treatment was implemented for ADHD treatment, with permitted changes to the implementation conditions. The study included screening (2–4 weeks), treatment (6 weeks), and follow‐up (4 weeks, SDT‐001 group only) periods, and tracking questionnaire survey through an investigator/subinvestigator up to 24 weeks after the last visit: with a total duration of 12–38 weeks for the SDT‐001 group and 8–34 weeks for the TAU group.

Participants entered the repetition part after completing the comparison part. The single‐group repetition part consisted of treatment (6 weeks) and follow‐up (12 weeks) periods and tracking questionnaire survey through an investigator/subinvestigator up to 24 weeks after the last visit: with a total duration of 18–42 weeks. During the treatment period, all participants received SDT‐001 once daily for 6 weeks with standard treatment. A treatment questionnaire was completed at the final visit or upon discontinuation. Participants who did not start ADHD pharmacotherapy underwent a tracking questionnaire up to 24 weeks after the last visit or on discontinuation of the repetition part. Participants using SDT‐001 throughout both comparison and repetition parts (SDT‐001/SDT‐001 or TAU/SDT‐001) had up to 56 weeks of follow‐up, including the tracking questionnaire to assess the need for ADHD pharmacotherapy post‐final visit.

### Randomization and blinding

Eligible participants were randomized (2:1) to the SDT‐001 or TAU group *via* stochastic minimization method at the enrollment center using the Interactive Web Response System. Allocation factors were prior ADHD pharmacotherapy (yes/no), age (≤12 and ≥13 years at enrollment), and ADHD type (combined, inattentive, or hyperactive–impulsive). The investigator delivered SDT‐001 (with code number and password) to participants on day 1 of the comparison (SDT‐001 group) and repetition parts.

### Study participants

Eligibility criteria are summarized in Supplementary Table S1. The comparison part included male or female outpatients aged 6–17 years, with confirmed ADHD diagnosis based on Diagnostic and Statistical Manual of Mental Disorders 5th edition and an ADHD‐RS‐I subscale score (physician's assessment) ≥15 at screening and baseline; received standard treatment for ADHD with sufficient effect; not received pharmacotherapy within 7 days before enrollment; and obtained acceptance from their teacher. The repetition part included participants from the comparison part who completed the last visit (end of the follow‐up for the SDT‐001 group and end of treatment for the TAU group). Participants from both parts were excluded for reasons given in Supplementary Table S1.

The study followed ethical principles, including the Declaration of Helsinki and Council for International Organizations of Medical Sciences International Ethical Guidelines, Ministerial Ordinance on Good Clinical Practice, and applicable laws and regulations. The study was approved by the Institutional Review Boards/Independent Ethics Committees (Supplementary Table S2). Written informed consent was obtained from participants and their legal representatives before study commencement.

### Study device

The details of the study device, SDT‐001, are summarized in Supplementary Methods.

### Study endpoints and assessments

#### Comparison part

Primary efficacy endpoint was change in ADHD‐RS‐I (physician's assessment) from baseline to week 6. Key secondary efficacy endpoints were changes in ADHD‐RS‐T and ADHD‐RS‐H (physician's assessment) from baseline to week 6. Other secondary efficacy endpoints included proportion of participants with 30% improvement in ADHD‐RS‐T, ADHD‐RS‐I, and ADHD‐RS‐H (physician's assessment); and changes in ADHD‐RS‐IV (teacher's assessment), Behavior Rating Inventory of Executive Function (BRIEF), Conners 3™ (parent's assessment) scale, Impairment Rating Scale (IRS), Pediatric Quality of Life Inventory (PedsQL™) Generic Core Scales, and EuroQol Five‐Dimensional Questionnaire, Youth version (EQ‐5D‐Y) scores from baseline to week 6.

#### Repetition part

The efficacy endpoints for the repetition part were changes from baseline in ADHD‐RS‐T, ADHD‐RS‐I, and ADHD‐RS‐H (physician's assessment); ADHD‐RS‐IV scores (teacher's assessment); BRIEF; Conners 3™ (parent's assessment) scale; IRS; PedsQL™ Generic Core Scales; and EQ‐5D‐Y scores.

The study endpoints were assessed using standardized rating scales and questionnaires, as described in Supplementary Methods.

### Treatment questionnaire survey and tracking questionnaire survey

The long‐term efficacy of SDT‐001 was assessed by patient transition to drug treatment. The treatment questionnaire survey was performed through an investigator/subinvestigator up to two times, at the final visit (or discontinuation) and at follow‐up if the participant proceeded to the tracking questionnaire. Participants in the comparison part who did not enter the repetition part completed the treatment questionnaire survey at week 10 (or discontinuation) for the SDT‐001 group and at week 6 (or discontinuation) for the TAU group. For the repetition part following the comparison part, the treatment questionnaire survey was performed at week 18 (or discontinuation). Participant eligibility for the tracking questionnaire survey was based on results of the treatment questionnaire survey/the end‐of‐treatment period questionnaire, which was administered after the final visit in the comparison part/week 18 (or discontinuation) upon initiation of ADHD treatment as needed, and standard treatment was continued at the request of the participant/guardian, although psychosocial treatment was needed, or if follow‐up was not possible.

### Safety assessments

The safety endpoints included adverse events (AEs), ADEs, Columbia‐Suicide Severity Rating Scale (C‐SSRS), and response to gaming addiction questionnaire, and were reported during each assessment. The study device‐related AEs were classified as ADEs. The C‐SSRS evaluated the presence or absence of suicidal ideation and behavior. Gaming disorder included the presence or absence of AEs related to gaming addiction assessed *via* a questionnaire on the desire to use the study device for extended periods and continuously, along with their reasons for these desires.

### Statistical analysis

#### Sample size

The target sample size for the comparison part was 150 participants (SDT‐001: 100; TAU: 50). The study was designed for a sample size of 135 evaluable participants (SDT‐001: 90; TAU: 45), with the difference between the two groups in ADHD‐RS‐I subscale scores assumed to be −2.5, with common standard deviation (SD) of 4.0, ensuring ≥90% power by two‐sample *t* test at a two‐sided significance level of 0.05, with a 2:1 randomization ratio.

Demographics and baseline characteristics were summarized using descriptive statistics, with categorical variables as numbers (n) and percentages (%), and continuous variables as mean and SD. Statistical tests were performed at a two‐sided significance level of 0.05, and all confidence intervals (CIs) estimated as two‐sided unless otherwise stated. All statistical analyses were performed using SAS (version 9.4) (SAS Institute, Cary, NC, USA).

#### Efficacy analyses

The efficacy analyses for both comparison and repetition parts were performed on the full analysis set (FAS). In the comparison part, the FAS included all participants randomized to the SDT‐001/TAU groups, who had ADHD‐RS‐I subscale score at baseline and ≥1 post‐baseline assessment timepoint. In the repetition part, the FAS included all participants who completed the comparison part and had an ADHD‐RS‐I subscale score at baseline and ≥1 post‐baseline assessment timepoint.

Primary endpoint, ADHD‐RS‐I (physician's assessment), and its difference with 95% CI between SDT‐001 and TAU groups at weeks 2, 4, and 6 were analyzed using a mixed‐effect model for repeated measures (MMRM) with unstructured covariance. The MMRM model included change from baseline as the response variable, with treatment group, assessment time, and their interaction as fixed effects. Covariates were baseline value, age (≤12 or ≥ 13 years), prior ADHD pharmacotherapy (yes/no), and ADHD type (combined, inattentive, or hyperactive–impulsive). Degrees of freedom were calculated using the Kenward–Roger method. The model estimated between‐group difference and its 95% CI at week 6 to test superiority of SDT‐001 over TAU. Key secondary endpoints, ADHD‐RS‐T and ADHD‐RS‐H (physician's assessment), were also analyzed using an MMRM model. To control the family‐wise type I error rate, a fixed‐sequence, gatekeeping multiple testing procedure was applied to the primary endpoint and key secondary endpoints. In the comparison part, the prespecified order for fixed sequence procedure was: (1) superiority of SDT‐001 to TAU in the primary endpoint, change from baseline in ADHD‐RS‐I (physician's assessment) at week 6, was tested at two‐sided significance level of 0.05; (2) only if a significant difference was detected in (1), the change from baseline in ADHD‐RS‐T (physician's assessment) at week 6 was tested; and (3) only if a significant difference was detected in (2), the change from baseline in ADHD‐RS‐H (physician's assessment) at week 6 was tested. For the primary and key secondary endpoints, subgroup analysis of covariance (ANCOVA) analyses was performed by age, sex, prior ADHD pharmacotherapy, and ADHD type.

Other secondary endpoints, IRS, PedsQL™ Generic Core Scales, and EQ‐5D‐Y (visual analog scale [VAS]) scores, were also analyzed using an MMRM analysis. Changes from baseline in ADHD‐RS‐IV (teacher's assessment), Conners 3™ (parent's assessment), and BRIEF scores were analyzed using ANCOVA, with baseline value, age, prior ADHD pharmacotherapy, and ADHD type as covariates. Additionally, for the proportion of participants with 30% improvements in ADHD‐RS‐IV (physician's assessment) scores, between‐group differences were estimated by the Cochran–Mantel–Haenszel method, stratified by age, prior ADHD pharmacotherapy, and ADHD type, with 95% CIs calculated.

In the repetition part, analyses were performed using descriptive statistics. The endpoints analyzed were the same as those for the comparison part. Changes from baseline were analyzed using the baseline data of the repetition part and assessment at day 1 of the comparison part. Change from baseline in ADHD‐RS‐IV (teacher's assessment) score was analyzed using the day 1 assessment of the comparison part as baseline since there was no assessment at day 1 of the repetition part.

#### Safety analyses

Safety analyses were performed in the safety analysis population. AEs reported after enrollment were used for safety analyses and classified by system organ class and preferred term using the Medical Dictionary for Regulatory Activities, version 24.1.

## Results

### Participant disposition

**Fig. 1:**
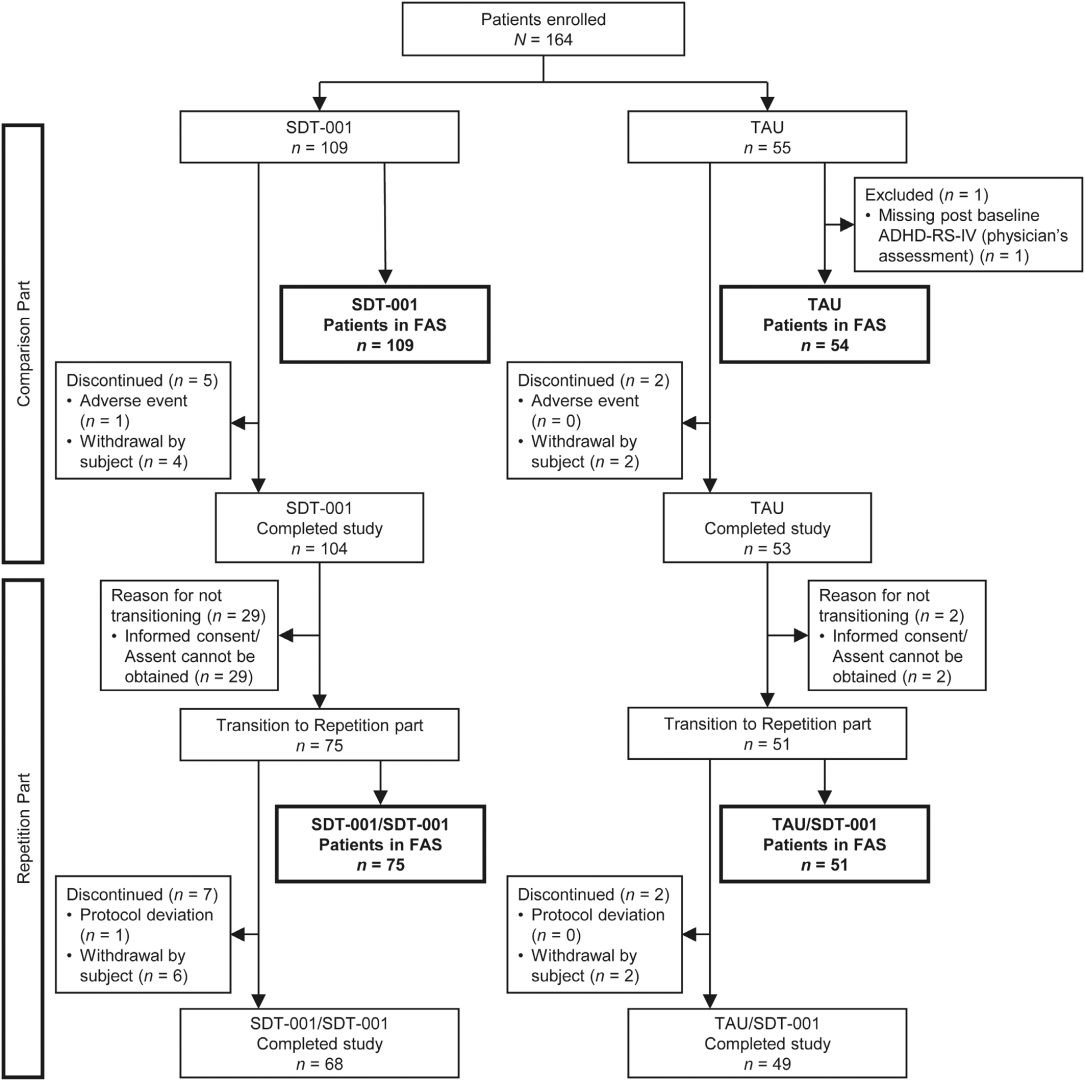
Participant disposition (CONSORT flowchart). ADHD‐RS‐IV, attention‐deficit/hyperactivity disorder rating scale IV; FAS, full analysis set; SDT‐001, investigational digital therapeutic; TAU, treatment as usual.

A total of 164 participants were enrolled and 157 completed the comparison part. Discontinuation in the SDT‐001 (*n* = 5, 4.6%) and the TAU (*n* = 2, 3.6%) groups occurred due to withdrawal (SDT‐001, *n* = 4; TAU, *n* = 2) or AEs (SDT‐001, *n* = 1). Of two participants who discontinued in the TAU group, one participant was excluded from the FAS due to missing post‐baseline ADHD‐RS‐IV (physician's assessment) score. Overall, 126 participants transitioned to the repetition part after completing the comparison part and 117 completed the repetition part. Nine participants discontinued, mostly due to participant withdrawal (Fig. 1).

### Participant demographic and baseline characteristics

There were no notable differences between treatment groups. In the SDT‐001 group, two participants changed their standard treatment during the study and received social skills training with environmental adjustments.

#### Comparison part

Participants' age (mean ± SD) and male sex were similar in both groups (SDT‐001, 9.8 ± 2.7 years and 74.3%; TAU, 9.5 ± 2.7 years and 74.1%, respectively). Baseline ADHD‐RS‐I subscale scores (mean ± SD) were 20.8 ± 3.3 in the SDT‐001 and 21.3 ± 3.3 in the TAU groups. Most had predominantly inattentive or combined ADHD type presentations, and all received psychosocial treatment, including environmental control (Table 1). Over half received only environmental control in both groups, whereas 14.7% in the SDT‐001 and 18.5% in the TAU groups received only psychosocial treatments. Prior ADHD pharmacotherapy was received by 27.5% in the SDT‐001 and 24.1% in the TAU groups.

**Table 1:** Demographics and baseline characteristics of participants (full analysis set) in the comparison and repetition parts

	Comparison part		Repetition part^†^	
	SDT‐001	TAU	SDT‐001/SDT‐001	TAU/SDT‐001
Characteristics	*n* = 109	*n* = 54	*n* = 75	*n* = 51
Sex				
Male	81 (74.3)	40 (74.1)	57 (76.0)	38 (74.5)
Female	28 (25.7)	14 (25.9)	18 (24.0)	13 (25.5)
Age (years)				
Mean ± SD	9.8 ± 2.7	9.5 ± 2.7	9.5 ± 2.7	9.4 ± 2.7
Median (min, max)	9.0 (6, 17)	9.0 (6, 17)	9.0 (6, 17)	9.0 (6, 17)
≥6 to ≤12 years	91 (83.5)	47 (87.0)	64 (85.3)	45 (88.2)
≥13 to ≤17 years	18 (16.5)	7 (13.0)	11 (14.7)	6 (11.8)
Race				
Asian	109 (100.0)	53 (98.1)	75 (100.0)	50 (98.0)
Other	0	1 (1.9)	0	1 (2.0)
Previous medication with indications for ADHD				
Yes	30 (27.5)	13 (24.1)	22 (29.3)	12 (23.5)
No	79 (72.5)	41 (75.9)	53 (70.7)	39 (76.5)
Psychosocial treatment including environmental control				
Yes	109 (100.0)	54 (100.0)	75 (100.0)	51 (100.0)
Type of psychosocial treatment				
Environmental control only	60 (55.0)	30 (55.6)	—	—
Psychosocial treatment other than environmental control	16 (14.7)	10 (18.5)	—	—
Both environmental control and psychosocial treatment	33 (30.3)	14 (25.9)	—	—
ADHD type				
Combined presentation	45 (41.3)	23 (42.6)	33 (44.0)	21 (41.2)
Predominantly inattentive presentation	64 (58.7)	31 (57.4)	42 (56.0)	30 (58.8)
Predominantly hyperactive–impulsive presentation	0	0	0	0
ADHD‐RS‐IV inattention subscale score (physician's assessment)				
Mean ± SD	20.8 ± 3.3	21.3 ± 3.3	20.7 ± 3.5	21.4 ± 3.3
Median (min, max)	20.0 (15, 27)	22.0 (15, 27)	20.0 (15, 27)	22.0 (16, 27)
<19	31 (28.4)	14 (25.9)	24 (32.0)	13 (25.5)
≥19	78 (71.6)	40 (74.1)	51 (68.0)	38 (74.5)

Data are presented as *n* (%) or mean ± SD unless specified.

ADHD, attention‐deficit/hyperactivity disorder; ADHD‐RS‐IV, attention‐deficit/hyperactivity disorder rating scale IV; max, maximum; min, minimum; SD, standard deviation; SDT‐001, investigational digital therapeutic; TAU, treatment as usual.

^†^ Based on values collected before the start of the comparison part.

#### Repetition part

Based on values collected before the comparison part initiated, participants' age (mean ± SD) was similar in both groups (SDT‐001/SDT‐001, 9.5 ± 2.7 years; TAU/SDT‐001, 9.4 ± 2.7 years), with more males (SDT‐001/SDT‐001, 76.0%; TAU/SDT‐001, 74.5%). Baseline ADHD‐RS‐I subscale score (mean ± SD) was 20.7 ± 3.5 in the SDT‐001/SDT‐001 and 21.4 ± 3.3 in the TAU/SDT‐001 groups, with similar ADHD presentations and psychosocial treatments (Table 1). Prior ADHD pharmacotherapy was received by 29.3% in the SDT‐001/SDT‐001 and 23.5% in the TAU/SDT‐001 groups.

### Device exposure and compliance rate

In the comparison part, the study device exposure (mean ± SD) in the SDT‐001 group was 35.3 ± 7.3 days; in the repetition part, it was 35.0 ± 8.6 days for SDT‐001/SDT‐001 and 34.6 ± 8.3 days for TAU/SDT‐001 groups. The compliance rate (mean ± SD) was 84.1 ± 16.1% in the SDT‐001 group, and 83.8 ± 18.0% in SDT‐001/SDT‐001 and 82.3 ± 18.8% in TAU/SDT‐001 groups, with most participants having ≥50% compliance (Supplementary Table S3).

### Efficacy

#### Comparison part

**Fig. 2:**
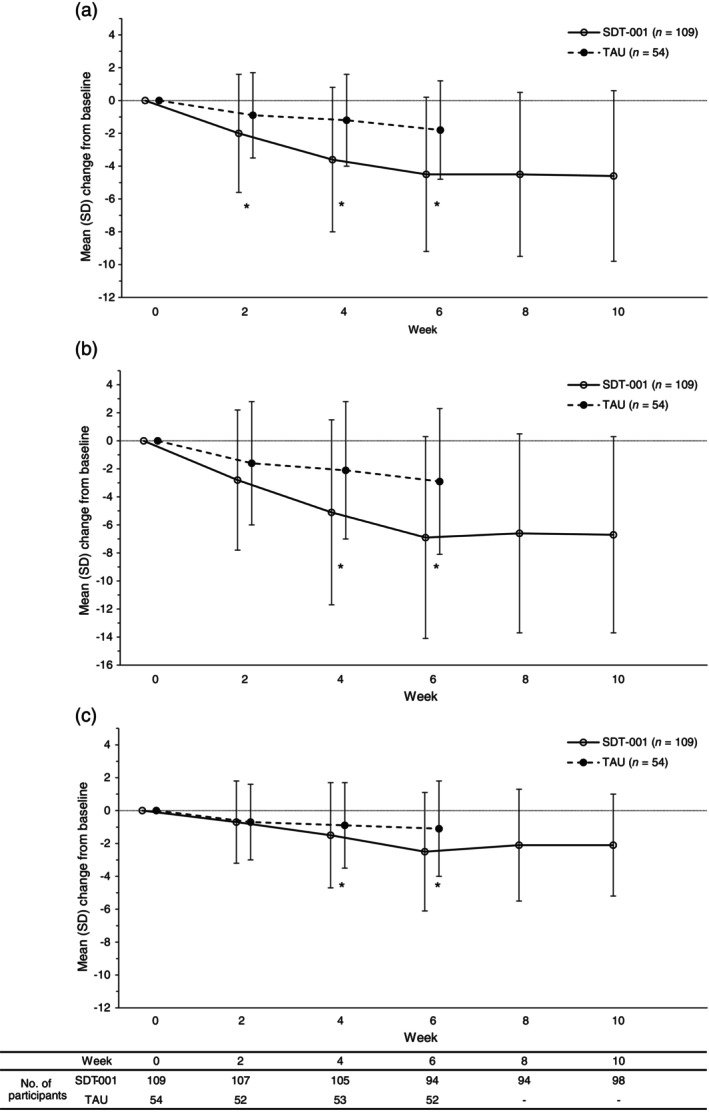
Change from baseline in ADHD‐RS‐IV (physicians' assessment) (a) inattention subscale score, (b) total score, and (c) hyperactive–impulsive subscale score during the treatment period and subsequent follow‐up period in the comparison part (FAS). *Statistical significance was determined with *P* < 0.05. A mixed‐effect model for repeated measures with unstructured covariance was applied to the change from baseline in score from week 2 to week 6 as response. This model was based on group, timepoint (week 2 to week 6) and interaction between group and timepoint as fixed effects, value at the baseline, binarized age group (≤12 years, ≥13 years), prior ADHD medication (yes/no), and ADHD type (combined, predominantly inattentive, predominantly hyperactive–impulsive) as covariates. ADHD‐RS‐IV, attention‐deficit/hyperactivity disorder rating scale IV; FAS, full analysis set; SD, standard deviation; SDT‐001, investigational digital therapeutic; TAU, treatment as usual.

Change from baseline in ADHD‐RS‐IV (physician's assessment) during the treatment and subsequent follow‐up is shown as Fig. 2a–c. Change from baseline to week 6 (adjusted mean ± standard error) in ADHD‐RS‐I (physician's assessment) subscale score was −4.44 ± 0.49 in SDT‐001 and −1.47 ± 0.65 in TAU groups. Adjusted mean change was significantly greater in the SDT‐001 than the TAU group (−2.97 [95% CI: −4.38, −1.56]; *P* < 0.0001 by MMRM analysis), demonstrating superiority of SDT‐001 over TAU, with improvement in ADHD‐RS‐I subscale score at 6 weeks' treatment. Additionally, change from baseline in ADHD‐RS‐T (physician's assessment) score (SDT‐001, −7.02 ± 0.74; TAU, −2.46 ± 0.99) was observed, with a between‐group difference of −4.56 (−6.75, −2.38; *P* < 0.0001 by MMRM analysis) at week 6. Change from baseline in ADHD‐RS‐H (physician's assessment) subscale score (SDT‐001, −2.57 ± 0.37; TAU, −1.02 ± 0.49) was also observed, with a between‐group difference of −1.55 (−2.64, −0.46; *P* = 0.0056 by MMRM analysis) at week 6 (Supplementary Table S4).

In the SDT‐001 group, reductions in scores were maintained even after the end of the treatment period and before initiation of the repetition treatment (between 6 and 10 weeks; Fig. 2a–c). In the subgroup ANCOVA analysis for ADHD‐RS‐IV: ADHD‐RS‐I, ADHD‐RS‐T, and ADHD‐RS‐H, although some subgroups were small, the adjusted mean differences for the change from baseline at week 6 favored SDT‐001 across all subgroups (Supplementary Fig. S2). Among the secondary endpoints, the degree of reduction in ADHD‐RS‐IV (teacher's assessment), BRIEF, Conners 3™ (parent's assessment) subscale, and IRS scores (greater reduction indicates improvement in symptoms) was greater in the SDT‐001 than TAU group. The degree of increase in PedsQL™ Generic Core Scales (greater increase indicates improvement in symptoms) was greater in the SDT‐001 than TAU group. The degree of increase in EQ‐5D‐Y (VAS) (greater increase indicates improvement in symptoms) was smaller in the SDT‐001 than TAU group (Supplementary Table S5). A significantly higher proportion of participants in the SDT‐001 group had >30% improvement from baseline (responders) in ADHD‐RS‐T (difference in proportion [95% CI], 25.3% [12.5, 38.1]; *P* = 0.0010), ADHD‐RS‐I (18.8% [6.5, 31.1]; *P* = 0.0092), and ADHD‐RS‐H (34.0% [19.6, 48.4]; *P* < 0.0001) scores at week 6 (Supplementary Table S6).

#### Repetition part

**Fig. 3:**
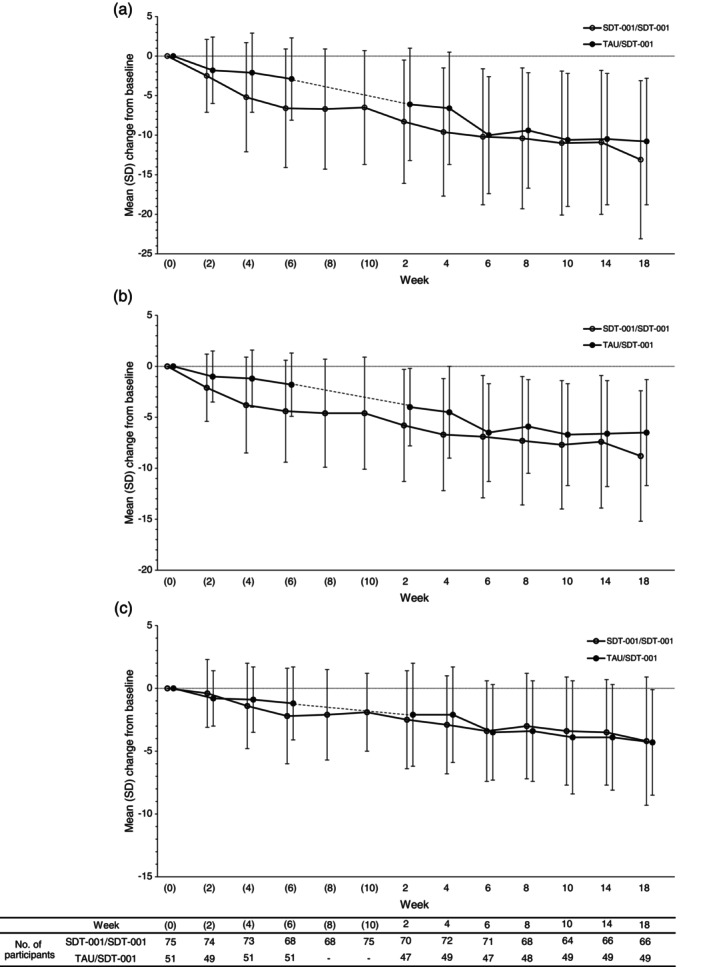
Change from baseline in ADHD‐RS‐IV (physician's assessment) (a) total score, (b) inattention subscale score, and (c) hyperactive–impulsive subscale score in the repetition part. ADHD‐RS‐IV, attention‐deficit/hyperactivity disorder rating scale IV; SD, standard deviation; SDT‐001, investigational digital therapeutic; TAU, treatment as usual.

Change from baseline in ADHD‐RS‐IV (physician's assessment) is shown as Fig. 3a–c. The SDT‐001/SDT‐001 and TAU/SDT‐001 groups showed consistent reductions in ADHD‐RS‐IV (physician's assessment) scores, which were maintained for 12 weeks without worsening.

Both SDT‐001/SDT‐001 and TAU/SDT‐001 groups showed consistent decrease in ADHD‐RS‐T (physician's assessment) scores from baseline throughout the follow‐up period. For the SDT‐001/SDT‐001 group, mean ± SD changes from baseline of the repetition part were −2.0 ± 4.4, −3.1 ± 4.1, and −3.7 ± 5.4 at weeks 2, 4, and 6, respectively, which consistently improved to −3.9 ± 5.5, −4.5 ± 5.7, −4.8 ± 5.9, and −5.9 ± 7.2 at weeks 8, 10, 14, and 18, respectively. The mean ± SD change from the comparison part baseline at week 2 in the repetition part (−8.3 ± 7.8) was more pronounced, reaching −13.1 ± 10.0 at week 18 in the repetition part. The TAU/SDT‐001 group also showed notable improvements in ADHD‐RS‐T (physician's assessment) scores, with mean ± SD change from the repetition part baseline of −3.1 ± 5.8, −3.6 ± 5.9, and −6.9 ± 6.5 at weeks 2, 4, and 6, respectively, and mean ± SD change from the comparison part baseline of −6.1 ± 7.1, −6.6 ± 7.1, and −10.0 ± 7.4 at weeks 2, 4, and 6 in the repetition part, which consistently improved, as in the SDT‐001/SDT‐001 group at week 18.

In the repetition part, both ADHD‐RS‐I and ADHD‐RS‐H (physician's assessment) subscale scores in the SDT‐001/SDT‐001 group consistently decreased from baseline throughout the treatment and follow‐up periods. Consistent improvements were also observed in the TAU/SDT‐001 group. The ADHD‐RS‐IV (teacher's assessment) scores decreased at week 6 of the repetition part compared to baseline in the comparison part in both SDT‐001/SDT‐001 and TAU/SDT‐001 groups (Supplementary Table S7).

### Results of tracking questionnaire survey

Among participants who completed comparison and repetition follow‐up periods, 54.7% (64/117) of those who used SDT‐001 once or twice (SDT‐001/SDT‐001, 60.3%; TAU/SDT‐001, 46.9%) did not begin to take ADHD medications at 24 weeks after initiation of follow‐up (Table 2).

**Table 2:** Treatment plan for ADHD at 24 weeks after initiation of follow‐up for study participants who completed the comparison and repetition follow‐up periods

	SDT‐001/SDT‐001	TAU/SDT‐001
	*n* = 68	*n* = 49
Begin to take medications with indications for ADHD^†^	*n* (%)	*n* (%)
Yes	23 (33.8)	25 (51.0)
Started medication for ADHD	17 (25.0)	17 (34.7)
Continue environmental control and/or psychosocial treatment (drug treatments are not an option due to participant/guardian preference)	6 (8.8)	8 (16.3)
No	45 (66.2)	24 (49.0)
Continue environmental control and/or psychosocial treatment (except when drug treatments are not an option due to participant/guardian preference)	41 (60.3)	23 (46.9)
Other^‡^	4 (5.9)	1 (2.0)

ADHD, attention‐deficit/hyperactivity disorder; SDT‐001, investigational digital therapeutic; TAU, treatment as usual.

^**†**^ If the results of the end‐of‐period or follow‐up questionnaire obtained 24 weeks after the start of the follow‐up survey were (i) to (iii), medication for ADHD was considered to have been started and counted. (i) If the participant answered “Started medications for ADHD” in the end‐of‐period questionnaire. (ii) If the participant answered “Due to participant/guardian preference, medications for ADHD was not an option” in the end‐of‐period questionnaire. (iii) If the participant answered “Continue environmental control and psychosocial treatment” in the follow‐up questionnaire (“Started medications for ADHD,” “Other,” or “Judged unable to follow‐up”). However, even if the participant answered “Continue environmental control and psychosocial treatment,” if the reason for choosing “Due to the participant/guardian preference, medications for ADHD was not an option,” it was considered that medication for ADHD had been started.

^‡^ Patients who selected “Other” in the end‐of‐period questionnaire and those who answered the follow‐up questionnaire in the above category (iii) but did not respond at 24 weeks after the start of the follow‐up survey were not considered to have started medications for ADHD at that time.

### Safety

In the comparison part, AEs were reported in 40.4% (44/109) participants in SDT‐001 and 18.2% (10/55) in TAU groups, with no “severe” events. Incidental infections were reported in 30.3% of participants in the SDT‐001 group. Common AEs in the SDT‐001 group of the comparison part were coronavirus disease‐2019 (11.9% [13/109]), nasopharyngitis (10.1% [11/109]), influenza (3.7% [4/109]), upper respiratory tract inflammation (2.8% [3/109]), and headache (2.8% [3/109]). In the TAU group, the most common AE was nasopharyngitis (3.6% [2/55]). In the repetition part, AEs were reported in 48.0% (36/75) participants in the SDT‐001/SDT‐001 and 52.9% (27/51) in the TAU/SDT‐001 groups, with similar common AEs in both groups (Table 3). No severe AEs were reported in the repetition part.

**Table 3:** Adverse events and adverse device events classified by system organ class, preferred term in each category

Comparison part		
	SDT‐001	TAU
	*n* = 109^‡^	*n* = 55^‡^
System organ class preferred term^†^	*n* (%)	*n* (%)
Participants with any AE	44 (40.4)	10 (18.2)
AE ≥2 either of SDT‐001 or TAU		
Infections and infestations	33 (30.3)	4 (7.3)
COVID‐19	13 (11.9)	1 (1.8)
Nasopharyngitis	11 (10.1)	2 (3.6)
Influenza	4 (3.7)	0
Gastroenteritis	2 (1.8)	0
Rhinitis	2 (1.8)	1 (1.8)
Upper respiratory tract infection	2 (1.8)	0
Nervous system disorders	3 (2.8)	0
Headache	3 (2.8)	0
Respiratory, thoracic, and mediastinal disorders	6 (5.5)	1 (1.8)
Upper respiratory tract inflammation	3 (2.8)	0
Rhinitis allergic	2 (1.8)	0
Participants with ADE ≥1	3 (2.8)	—
Psychiatric disorders	1 (0.9)	—
Frustration tolerance decreased	1 (0.9)	—
Nervous system disorders	1 (0.9)	—
Headache	1 (0.9)	—
Gastrointestinal disorders	1 (0.9)	—
Nausea	1 (0.9)	—

Repetition part		
	SDT‐001/SDT‐001	TAU/SDT‐001
	*n* = 75^‡^	*n* = 51^‡^
System organ class preferred term^†^	*n* (%)	*n* (%)
Participants with any AE	36 (48.0)	27 (52.9)
AE ≥2 either of SDT‐001 or TAU		
Infections and infestations	22 (29.3)	17 (33.3)
Nasopharyngitis	7 (9.3)	10 (19.6)
COVID‐19	7 (9.3)	6 (11.8)
Gastroenteritis	2 (2.7)	2 (3.9)
Influenza	2 (2.7)	1 (2.0)
Pharyngitis	2 (2.7)	0
Immune system disorders	2 (2.7)	1 (2.0)
Seasonal allergy	2 (2.7)	1 (2.0)
Nervous system disorders	2 (2.7)	1 (2.0)
Respiratory, thoracic, and mediastinal disorders	7 (9.3)	3 (5.9)
Rhinorrhea	2 (2.7)	1 (2.0)
Upper respiratory tract inflammation	3 (4.0)	0
Rhinitis allergic	2 (2.7)	0
Gastrointestinal disorders	5 (6.7)	4 (7.8)
Abdominal pain	2 (2.7)	1 (2.0)
Vomiting	0	2 (3.9)
Skin and subcutaneous tissue disorders	1 (1.3)	3 (5.9)
General disorders and administration site conditions	4 (5.3)	3 (5.9)
Pyrexia	4 (5.3)	3 (5.9)
Injury, poisoning, and procedural complications	7 (9.3)	2 (3.9)
Ligament sprain	2 (2.7)	0
Contusion	2 (2.7)	0
Patients with ADE ≥1	0	1 (2.0)
Gastrointestinal disorders	0	1 (2.0)
Vomiting	0	1 (2.0)

ADE is defined as an AE with a relationship to the study device that is assessed as “related.”

^†^ System organ class and preferred term of the Medical Dictionary for Regulatory Activities version 24.1.

^‡^ The safety analysis population included all participants who were randomized to the SDT‐001 group and used the study device at least once, all participants randomized to the TAU group, and all participants who entered the repetition part after the comparison part and used the study device at least once.

ADE, adverse device event; AE, adverse event; COVID‐19, coronavirus disease; SDT‐001, investigational digital therapeutic; TAU, treatment as usual.

In the comparison part, ADEs in the SDT‐001 group included one case each of nausea, headache, and decreased frustration tolerance, which were mild and recovered/resolved. No ADEs were observed in the TAU group. In the repetition part, no ADEs were observed in the SDT‐001/SDT‐001 group. One ADE (vomiting) was reported in the TAU/SDT‐001 group (2.0% [1/51]), which was mild and recovered/resolved (Table 3).

No fatal/nonfatal serious AEs were reported in the comparison part. In the repetition part, one moderate nonfatal serious AE (cyclic vomiting syndrome) was reported in the SDT‐001/SDT‐001 group during the follow‐up period, approximately 1 month after study device use ended. The event resolved after 3 days' hospitalization and was considered unrelated to the study device by the investigator. In the comparison part, one participant in the SDT‐001 group experienced a mild ADE (decreased frustration tolerance) leading to discontinuation, which resolved without treatment. No AEs leading to discontinuation were reported in the repetition part.

In neither part of the study did any participants in the SDT‐001 or TAU groups exhibit a shift to a higher suicidal risk, and no suicidal ideation or behavior was observed (per C‐SSRS). The gaming disorder questionnaire also showed no trend towards gaming disorder after using SDT‐001, either initially or after an additional cycle.

## Discussion

This phase 3 study assessed the efficacy and safety of SDT‐001 *versus* TAU in Japanese children and adolescents with ADHD. The findings showed superiority of SDT‐001 over TAU, with a significant reduction from baseline in ADHD‐RS‐I (physician's assessment) subscale score at week 6 (*P* < 0.0001) in the comparison part. This aligns with a previous study of AKL‐T01 (STARS‐ADHD), which showed improvements in TOVA‐API scores after a single 4‐week treatment regimen.^19^ In addition, at week 6, the reductions in ADHD‐RS‐T (*P* < 0.0001) and ADHD‐RS‐H (*P* = 0.0056) scores from baseline were significantly greater with SDT‐001 than TAU. Moreover, reductions in scores were maintained after treatment discontinuation (week 10) and before initiation of the repetition part. Additionally, ADHD‐RS‐IV (teacher's assessment), BRIEF, Conners 3™ (parent's assessment) subscale, IRS, and PedsQL™ Generic Core Scale scores consistently improved with SDT‐001 *versus* TAU. In this study, subgroup analysis showed that change from baseline in each ADHD‐RS‐IV (physician's assessment) score at week 6 favored SDT‐001 *versus* TAU groups, regardless of prior medications for ADHD, age, sex, or ADHD type.

In the repetition part, both SDT‐001/SDT‐001 and TAU/SDT‐001 groups showed consistent reduction in ADHD‐RS‐IV (physician's assessment) scores from baseline to week 6, which were maintained up to 12 weeks after treatment completion. These findings revealed a tendency for participants to exhibit improvement in ADHD symptoms with SDT‐001 for 6 weeks.

The SDT‐001 showed consistently greater improvements across all endpoints at week 6 (end of treatment in both parts), with reductions in the scores maintained for up to 12 weeks after treatment completion: evaluation of the effectiveness of three or more repeated cycles (SDT‐001/SDT‐001/SDT‐001) could be a future prospect.

Pharmacotherapy improves ADHD symptoms, but these often recur upon drug discontinuation.^27^, ^28^, ^29^, ^30^, ^31^ Persistent reduction in scores after treatment cessation, similar to the prior phase 2 findings,^25^ and 54.7% of participants in SDT‐001/SDT‐001 and TAU/SDT‐001 groups not requiring drug therapy 24 weeks after initiation of follow‐up, suggest that SDT‐001 might provide additional long‐term treatment benefits.

AEs reported were consistent with the previous phase 2 study of SDT‐001,^25^ with no new safety concerns, severe AEs, or signs of dependence/addiction. ADHD pharmacotherapy is commonly associated with AEs like nausea, vomiting, loss of appetite, insomnia, depressive symptoms, irritability, and social withdrawal,^32^ while SDT‐001 was well‐tolerated. The SDT‐001 group had a higher incidence of infections than the TAU group; however, it was similar to or lower than those in ADHD pharmacotherapy trials in children with ADHD.^33^, ^34^, ^35^ Moreover, the reported infections were incidental and unrelated to SDT‐001.

Therapies such as environmental adjustment and psychosocial treatment are recommended over drug therapy for ADHD; however, there is a lack of specialized institutions and medical professionals capable of providing appropriate treatments. Psychosocial treatment may be safer, but outcomes may be variable owing to therapist/clinician variability^14^, ^16^; SDT‐001, however, could have negligible clinician‐induced instability/variability in treatment responses and outcomes. Further, the compliance rate with SDT‐001 exceeded 80%, which was higher than the reported rates with pharmacotherapy (67.5%) and psychostimulants (74.2%) in children and adolescents (6–17 years) with ADHD,^36^, ^37^ suggesting that SDT‐001 could be a suitable option for patients who cannot undergo pharmacotherapy. Furthermore, a critical finding of the prior pediatric trials with AKL‐T01 and SDT‐001 is reflected in this phase 3 study of SDT‐001: SDT‐001 appears to be effective in improving ADHD symptoms when added to TAU.^25^ Hence, SDT‐001 could be an option in multimodal therapy approach in managing ADHD symptoms and functional impairment, as suggested in Japanese clinical guidelines.^7^, ^8^ Future studies should evaluate potential differences in SDT‐001 treatment outcomes according to concomitant/stimulant medication status and timing relative to SDT‐001 engagement.

Dual‐task training activates right‐sided network,^38^ whereas task‐switching training improves inhibitory control and verbal working memory.^39^ Dual‐task training also activates primary motor cortex and supplementary motor area, responsible for planning, control, and execution of movements.^40^ Moreover, both focused and divided attention tasks engage a widespread, predominantly right‐sided network involving prefrontal and parietal structures, with increased activity and recruitment of left‐sided homologues under higher cognitive demands.^38^ Hence, SDT‐001, being a dual‐task device, likely involves activation of neuronal substrates engaged in cognitive–motor interactions, with differences in activation patterns arising from varying demands of executive control and with increase in task difficulty,^38^, ^40^ potentially modulating neural activity to enhance cognitive–motor performance. Elucidation of the mechanisms underlying this effect is needed.

We circumvented some major limitations of cognitive training studies (smaller sample size, short duration of treatment, and follow‐up^19^, ^41^) in this study by including a larger sample size and a 52‐week follow‐up throughout both the comparison and repetition parts. The study design was limited to assessing the efficacy of SDT‐001 *versus* TAU in an open‐labeled/non‐blinded treatment assignment; however, it was rationalized based on results from the previous phase 2 study.

## Conclusion

In this phase 3 study, SDT‐001 demonstrated a significant improvement in inattention and hyperactivity‐impulsivity symptoms in Japanese children and adolescents with ADHD. Reductions in ADHD‐RS‐IV scores were maintained up to 12 weeks after completion of the 6‐week treatment. Furthermore, SDT‐001 consistently showed greater improvement in ADHD‐RS‐IV scores (from baseline to week 6), after both one and two treatment cycles. These findings, and its safety profile, suggest that SDT‐001 could offer a novel and effective digital treatment option, addressing the limitations associated with traditional psychosocial treatments and pharmacotherapy, for the management of ADHD. Future research could explore the mechanisms underlying its sustained benefit even after usage discontinuation and the potential integration of efficacy with existing treatment protocols.

## Author contributions

KM, TM, RN, and TO conceptualized, designed, and interpreted the results. HF conceptualized, designed, acquired, and analyzed the data. NK and YK conceptualized, designed, and analyzed the data. All authors were involved in drafting and/or critically reviewing the manuscript and gave final approval of the version to be published. All authors agree to be accountable for all aspects of the work.

## Funding

This study was funded by Shionogi & Co., Ltd., Japan.

## Disclosure statement

KM has received financial support from Shionogi & Co., Ltd.; honoraria from Shionogi & Co., Ltd., Sumitomo Pharma Co., Ltd., and Takeda Pharmaceutical Co., Ltd.; travel and accommodation expenses from Otsuka Pharmaceutical Co., Ltd.; travel and accommodation expenses for the spouse from Pfizer; and a consulting fee from Shionogi & Co., Ltd., EA Pharma Co., Ltd., Sumitomo Pharma Co., Ltd., and Otsuka Pharmaceutical Co., Ltd. TM has received payment or honoraria for lectures, presentations, speakers' bureaus, manuscript writing, or educational events from Nobelpharma Co., Ltd., Janssen Pharmaceutical K.K., and Shionogi & Co., Ltd.; and consulting fees from Shionogi & Co., Ltd. RN declares no financial competing interests. NK, YK, and HF are employees of Shionogi & Co., Ltd. and may hold stocks in the company. TO has received payment or honoraria for lectures, presentations, speakers' bureaus, manuscript writing, or educational events from Janssen Pharmaceutical K.K., Shionogi & Co., Ltd., Yoshitomi Yakuhin Co., Takeda Pharmaceutical Co., Ltd., and Nobelpharma Co., Ltd. TO is an Editorial Board member of Psychiatry and Clinical Neurosciences and a co‐author of this article. To minimize bias, TO was excluded from all editorial decision‐making related to the acceptance of this article for publication.

## Supporting information


**Figure S1.** Study design.
**Figure S2.** Subgroup analysis of change from baseline in ADHD‐RS‐IV (physician's assessment) (a) ADHD‐RS‐I (b) ADHD‐RS‐T (c) ADHD‐RS‐H scores in comparison part at week 6 (FAS).
**Table S1.** Study eligibility criteria for participation.
**Table S2.** List of Institutional Review Boards (IRBs).
**Table S3.** Study device exposure and compliance.
**Table S4.** Change from baseline in ADHD‐RS‐IV (physicians' assessment) at week 6 in comparison part.
**Table S5.** Summary of secondary endpoints at week 6 in comparison part.
**Table S6.** Proportion of participants with more than 30% improvement in ADHD‐RS‐IV (physician's assessment) score at week 6 in comparison part.
**Table S7.** Change from baseline in ADHD‐RS‐IV (teacher's assessment) scores in repetition part.
