# Supplementary material for: Efficacy and safety of SDT‐001, a dual‐task digital device, in managing attention‐deficit/hyperactivity disorder symptoms in children and adolescents: a phase 3, randomized, standard treatment‐controlled study
Source: Psychiatry Clin Neurosci. 2025 May 2;79(8):447–57. doi: 10.1111/pcn.13833 (PMC12319664; doi:10.1111/pcn.13833)
Supplement: Supplementary file 1 — Figure S1. Study design. Figure S2. Subgroup analysis of change from baseline in ADHD‐RS‐IV (physician's assessment) (a) ADHD‐RS‐I (b) ADHD‐RS‐T (c) ADHD‐RS‐H scores in comparison part at week 6 (FAS). Table S1. Study eligibility criteria for participation. Table S2. List of Institutional Review Boards (IRBs). Table S3. Study device exposure and compliance. Table S4. Change from baseline in ADHD‐RS‐IV (physicians' assessment) at week 6 in comparison part. Table S5. Summary of secondary endpoints at week 6 in comparison part. Table S6. Proportion of participants with more than 30% improvement in ADHD‐RS‐IV (physician's assessment) score at week 6 in comparison part. Table S7. Change from baseline in ADHD‐RS‐IV (teacher's assessment) scores in repetition part. [file PCN-79-447-s001.docx]

**SUPPLEMENTARY MATERIAL**

**Supplementary Methods**

**Study device**

The study device, SDT-001, by Akili Interactive Labs, Inc. (MA, USA) is a software program (application) functioning as a medical device, featuring a dual-task mechanism (performing 2 tasks simultaneously) and allowing task difficulty adjustment based on each participant’s achievement level. SDT-001 was an investigational digital therapeutic using a proprietary algorithm to improve attention and ADHD symptoms through personalized difficulty levels. Details of SDT-001 (AKL-T01) were previously published by Kollins et al. (2021)^1^. SDT-001 was used once daily (approximately 25 minutes) for 6 weeks in both the comparison and repetition parts. SDT-001 was pre-installed on an iPad mini^®^ tablet (Apple, Japan) and provided to the study sites.

Reference

1. Kollins SH, Childress A, Heusser AC, Lutz J. Effectiveness of a digital therapeutic as adjunct to treatment with medication in pediatric ADHD. NPJ Digit. Med. 2021; **4**: 58.

**Assessment of efficacy endpoints**

To assess ADHD symptoms and their impact on children, several standardized rating scales and questionnaires are utilized, including the Attention-Deficit/Hyperactivity Disorder Rating Scale IV (ADHD-RS-IV), Behavior Rating Inventory of Executive Function (BRIEF), Parent-rated Conners 3™, Impairment Rating Scale (IRS), Pediatric Quality of Life Inventory (PedsQL™), and EuroQol Five-Dimensional Questionnaire, Youth version (EQ-5D-Y).

- The ADHD-RS-IV is an 18-item assessment for core symptoms of ADHD. ADHD 18 diagnostic criteria (the odd criteria for inattention, and the even criteria for hyperactivity-impulsivity) to be rated on 4-point rating scale (0=never/rarely, 1=sometimes, 2=often, and 3=very often).^1,2^
- The BRIEF is a rating scale used to assess daily activities on a child’s executive function.^3,4^
- The Conners 3™ is a 110-item assessment scale used by parents to assess ADHD-related symptoms.^5^
- The IRS is an 8-item assessment for functional impairment, evaluating the impact on a child’s functioning across a range of domains via a visual analog scale (VAS).^6^
- The PedsQL™ is a 23-item measure for assessing health-related quality of life in children.^7^
- The EQ-5D-Y measures health-related quality of life in children and consists of a descriptive questionnaire and the EuroQol (EQ) VAS score.^8^

**References**

1. Kollins SH, DeLoss DJ, Cañadas E et al. A novel digital intervention for actively reducing severity of paediatric ADHD (STARS-ADHD): a randomised controlled trial. Lancet Digit. Health 2020; **2**: e168–e178.
2. DuPaul G, Power T, Anastopoulos A, Reid R. *ADHD Rating Scale-IV: Checklists, Norms, and Clinical Interpretation*. Guilford Press, New York, 1998.
3. Gioia GA, Isquith PK, Guy SC, Kenworthy L. Behavior Rating Inventory of Executive Function^®^, Second Edition (BRIEF®2). Lutz, Fl; PAR Inc. 2015.
4. Mullins D, Pfefferbaum B, Schultz H, Overall JE. Brief psychiatric rating scale for children: quantitative scoring of medical records. *Psychiatry Res*. 1986; **19**: 43–49.
5. Conners CK. Conners 3rd Edition™ manual. New York, Multi-Health Systems Inc, 2008.
6. Fabiano GA, Pelham WE Jr, Waschbusch DA *et al*. A practical measure of impairment: psychometric properties of the impairment rating scale in samples of children with attention deficit hyperactivity disorder and two school-based samples. *J. Clin. Child Adolesc. Psychol.* 2006; **35**: 369–385.
7. Varni JW, Burwinkle TM, Seid M, Skarr D. The PedsQL 4.0 as a pediatric population health measure: feasibility, reliability, and validity. *Ambul. Pediatr*. 2003; **3**: 329–341.
8. EuroQol Research Foundation. EQ-5D-Y User Guide. 2020. Available at <https://euroqol.org/publications/user-guides>. [Accessed 30 Sept 2024]

**Supplementary Figures**

**Supplementary Fig. S1** Study design.


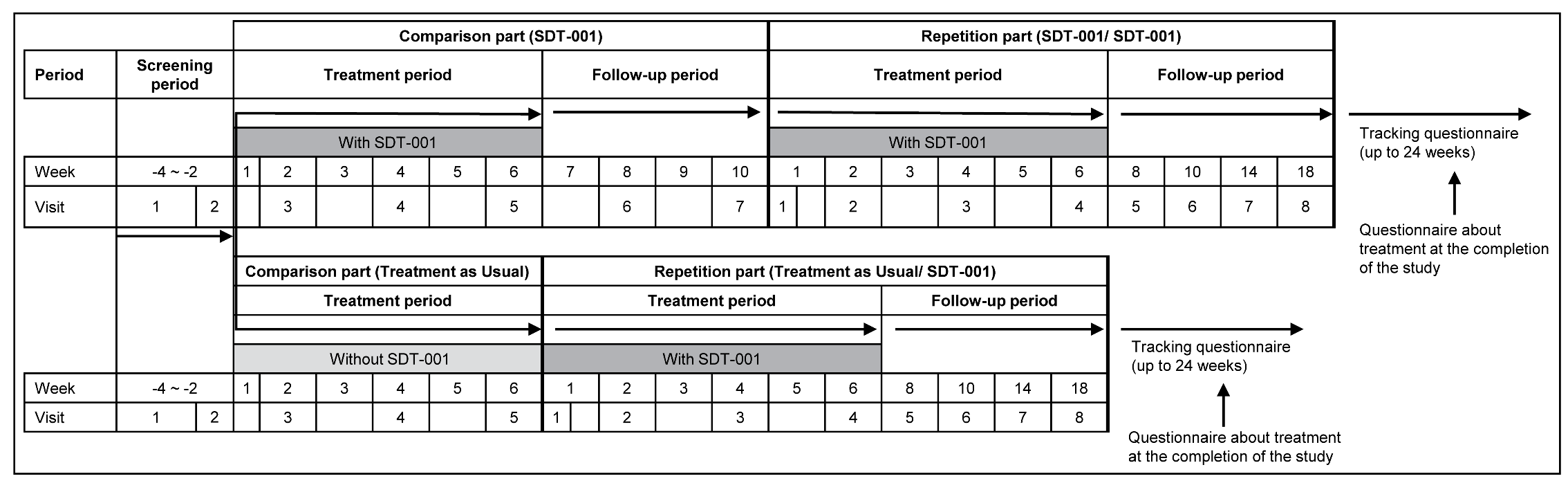


SDT-001, investigational digital therapeutic; TAU, treatment as usual.

**Supplementary Fig. S2** Subgroup analysis of change from baseline in ADHD-RS-IV (physician’s assessment) (a) ADHD-RS-I (b) ADHD-RS-T (c) ADHD-RS-H scores in comparison part at week 6 (FAS).


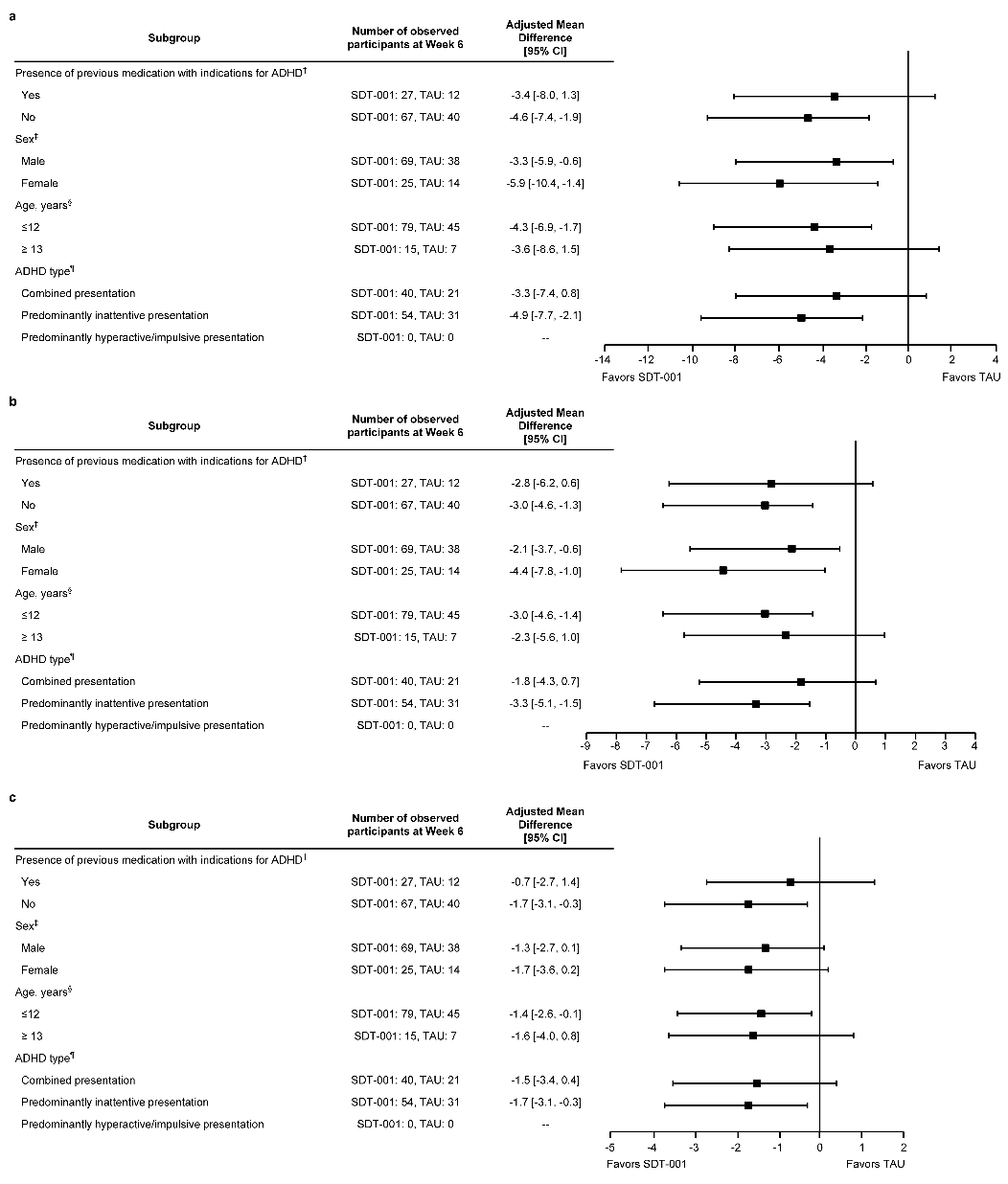


Analysis of covariance was performed on the change at week 6 from baseline; it is based on group as fixed effects, covariates are listed below

^†^Covariates for presence of previous medication for ADHD: baseline value, age category, ADHD type.

^‡^Covariates for sex: baseline value, age category, ADHD type, presence of previous medication with indications for ADHD.

^§^Covariates for age: baseline value, ADHD type, presence of previous medication with indications for ADHD.

^¶^Covariates for ADHD type: baseline value, age category, presence of previous medication with indications for ADHD.

ADHD, attention-deficit/hyperactivity disorder; ADHD-RS-IV, attention-deficit/hyperactivity disorder rating scale IV; CI, confidence interval; FAS, full analysis set; SDT-001, investigational digital therapeutic; TAU, treatment as usual.

**Supplementary Tables**

**Supplementary Table S1** Study eligibility criteria for participation

| **Comparison part** | |
| --- | --- |
| Inclusion criteria | Patients meeting all the following inclusion criteria were eligible for the study |
| Age | 1. Outpatients who were pupils or students aged 6–17 years at the time of informed consent |
| Study population and disease characteristics | 1. Patients whose primary diagnosis^†^ according to the diagnostic criteria of the DSM-5 was ADHD:  - 314.01 (F90.2) Combined - 314.00 (F90.0) Predominantly inattentive - 314.01 (F90.1) Predominantly hyperactive-impulsive |
|  | 1. Patients who were confirmed to have received environmental control and/or psychosocial treatment for ADHD for a sufficient period at the time of informed consent and were considered unlikely to have a sufficient effect. |
|  | 1. Patients who had not received pharmacotherapy for ADHD within 7 days before informed consent. |
|  | 1. Patients whose ADHD-RS-IV (physician’s assessment) inattentive subscale scores at both visit 1 and visit 2 were 15 points or higher. |
|  | 1. A patient who could obtain acceptance from his/her teacher to perform assessments and for whom the results of teacher’s assessments could be confirmed at day one.   ^†^ Disease which was the main factor requiring outpatient medical care in patients with more than one diagnosis made in the outpatient setting. |
| Sex | 1. Male and female |
| Informed consent | 1. Written consent to voluntary study participation could be obtained from the patient’s legally authorized representative. For patients aged 13 years or older, written informed assent was also to be obtained from patients themselves. For patients aged 6 to <13 years, written informed assent was to be obtained from patients themselves wherever possible. |
| Exclusion criteria | Patients meeting any of the following criteria were ineligible for the study |
| Medical conditions | 1. Patients with psychiatric disease such as schizophrenia spectrum disorder, depression, or bipolar disorder. However, patients with concurrent autism spectrum disorder or localized learning disorder might have been included. |
|  | 1. Patients with personality disorder or intellectual disability, or patients with suspected intellectual disability with an intelligence quotient of <70 in an intelligence test (or a previous intelligence test within the past 1 year, if any, and if acceptable in the opinion of the investigator/subinvestigator). |
|  | 1. Patients concurrently or previously with convulsion or severe tic disorder (including Tourette’s disorder). However, patients concurrently or previously with febrile convulsion were eligible. |
| Prior/concomitant therapy^‡^ | 1. Patients who had used any prohibited concomitant drugs/therapies during the period from the time of informed consent to day one. |
| Prior/concurrent clinical study experience | 1. Patients who had received any other investigational drug or used any other study device within 90 days prior to visit 1. |
|  | 1. Patients who had participated in this study or any of other studies of the study device. |
| Diagnostic assessments | 1. Patients whose percent change in ADHD-RS-IV (physician’s assessment) inattentive subscale score at day one exceeded 30% compared to that at visit 1. |
|  | 1. Patients who were unable to operate the application for physical or other reasons (eg, deafness, color blindness, broken hands, or arms, etc.). |
|  | 1. Patients with suspected gaming disorder (playing games had greatly interfered with everyday life [eg, school life, sleep, etc.]). |
|  | 1. Patients with suicidal tendency meeting any of the followings:  - A patient previously with suicide attempts. - A patient who had answered “Yes” to Question 4 or Question 5 regarding suicidal ideation or any questions regarding suicidal behavior in the C-SSRS within the past 6 months. |
|  | 1. Patients with suspected substance-related disorders within 180 days prior to visit 1. |
| Other exclusion criteria | 1. Other patients who were considered by the investigator/subinvestigator to be inappropriate for participation in the study, such as those who had difficulty in completing the study or making assessments, those who might have been disadvantaged by participation in the study, or those for whom it was difficult to ensure safety during the study period. |
| **Repetition part** | |
| Inclusion criteria | Patients meeting all the following inclusion criteria were eligible for the study |
| Age | 1. Outpatients who were school children or students aged 6–17 years before initiating the treatment period of repetition part (visit 1). |
| Study population and disease characteristics | 1. Patients who had participated in the preceding comparison part and who had completed the last visit (at the end of follow-up period for the SDT-001 group and at the end of treatment period for the TAU group). |
|  | 1. A patient who could obtain acceptance from his/her teacher to continue performing assessments. |
| Sex | 1. Male and female |
| Informed consent | 1. Written consent to voluntary study participation could be obtained from the patient’s legally authorized representative. For patients aged 13 years or older, written informed assent was also to be obtained from patients themselves. For patients aged 6 to <13 years, written informed assent was to be obtained from patients themselves wherever possible. |
| Exclusion criteria | Patients meeting any of the following criteria were ineligible for the repetition part. |
| Medical conditions | 1. Patients who were unable to operate the application for physical and other reasons (eg, deafness, color blindness, broken hands, or arms, etc.). |
|  | 1. Patients who were considered by the investigator/subinvestigator to be inappropriate for participation in the repetition part due to AEs that occurred during the precedent comparison part and were unresolved before initiating the treatment period of repetition part (visit 1). |
| Prior/Concomitant therapy^‡^ | 1. Patients who had used any prohibited concomitant drugs during the study period (from visit 1 to the end of follow-up period) before initiating the treatment period of repetition part (visit 1). |
| Other exclusion criteria | 1. Patients who had answered “Yes” to Question 4 or Question 5 regarding suicidal ideation or any questions regarding suicidal behavior in the C-SSRS before initiating the treatment period of repetition part (visit 1). |
|  | 1. Other patients who were considered by the investigator/subinvestigator to be inappropriate for participation in the study, such as those who had difficulty in completing the study or making assessments, those who might have been disadvantaged by participation in the study, or those for whom it was difficult to ensure safety during the study period. |
| **Definitions** | |
| Prior therapy | Therapies which were provided before informed consent in the comparison part.  Therapies included medications for ADHD used before informed consent (regardless of indication for ADHD), and psychosocial treatments for ADHD (including environmental adjustments) used within 6 months before informed consent for the comparison part. |
| Concomitant therapy | Therapies (including prescription drugs, vaccines, over-the-counter drugs, and health foods) provided from the time of informed consent for the comparison part to the last visit of the repetition part (at the end of follow-up period or discontinuation).  For participants who have not entered the repetition part, concomitant therapies are defined as therapies provided by the final assessment of the comparison part. |
| Prohibited concomitant therapy | The use of the following drugs (including prescription drugs, OTC drugs, and health foods) and therapies was prohibited from the time of informed consent to visit 7 or discontinuation (treatment period or follow-up period); however, drugs other than the investigational drug that were intended for external use or eye drops for local action might be used concomitantly   - Antipsychotic drugs - Selective noradrenaline reuptake inhibitors (e.g., atomoxetine hydrochloride) - Selective α2A adrenoceptor agonist (e.g., guanfacine hydrochloride) - Stimulants (e.g., methylphenidate hydrochloride, lisdexamfetamine mesilate) and antitussive drugs and common cold drugs that contain such components - Depressants (e.g., sedatives, barbiturates) - Antidepressants - Mood stabilizers - Antianxiety drugs - Benzodiazepines - Appetite suppressants - Monoamine oxidase inhibitors - Anticonvulsants - Sedative antihistamines (including drugs containing sedative antihistamines) - Hypnotics - Other investigational drug/investigational device - Nutraceuticals that may have effects on the central nervous system (e.g., St. John’s wort, ginkgo leaf, kava, ephedra, melatonin) |

^‡^General name of WHO Drug Dictionary version September 2021. If a participant gets the same medical treatment more than once, the participant will be counted once.

ADHD, attention-deficit/hyperactivity disorder; ADHD-RS-IV, attention-deficit/hyperactivity disorder rating scale IV; C-SSRS, Columbia-Suicide Severity Rating Scale; DSM-5, Diagnostic and Statistical Manual of Mental Disorders, fifth edition; OTC, over-the-counter.

**Supplementary Table S2** List of Institutional Review Boards (IRBs)

| **Study Center Name** | **Name of IRB / IEC** |
| --- | --- |
| Hokkaido University Hospital | Hokkaido University Hospital Institutional Review Board |
| Ujiie Memorial Clinic for Children | Sugiura Clinic Institutional Review Board |
| Kon Pediatric Clinic | Sugiura Clinic Institutional Review Board |
| Igarashi Pediatrics Clinic | Sugiura Clinic Institutional Review Board |
| Nanko kokorono Clinic | Nanko kokorono Clinic Institutional Review Board |
| Hitachinaka General Hospital | Review Board of Human Rights and Ethics for Clinical Studies Institutional Review Board |
| Tsuchiura Kyodo General Hospital | Review Board of Human Rights and Ethics for Clinical Studies Institutional Review Board |
| Ibaraki Children’s Hospital | Sugiura Clinic Institutional Review Board |
| Ken Clinic | Mizuo Clinic Institutional Review Board |
| Fukaya Mental Clinic | Shoda Hospital Institutional Review Board |
| Hello Clinic | Sugiura Clinic Institutional Review Board |
| Kohnodai Hospital,National Center for Global Health and Medicine | Kohnodai Hospital,National Center for Global Health and Medicine Institutional Review Board |
| Sotobou Kodomo Clinic | Dr. Mano Medical Clinic Institutional Review Board |
| Tokyo Women's Medical University Hospital | Tokyo Women's Medical University Institution Review Board |
| National Center of Neurology and Psychiatry | National Center of Neurology and Psychiatry Institutional Review Board |
| Kyorin University Hospital | Kyorin University Hospital Institutional Review Board |
| Shimada Ryoiku Medical Center for Challenged Children | Japan Conference of Clinical Research Institutional Review Board |
| Minami-aoyama Antique Street Clinic | Suzuki Internal & Circulatory Medical Clinic Institutional Review Board |
| Tokyo Nishi Tokushukai Hospital | Tokushukai Group Institutional Review Board |
| Oyamadai Suku-suku Clinic | Mizuo Clinic Institutional Review Board |
| Hattatsu Shinryo Clinic | Japan Conference of Clinical Research Institutional Review Board |
| Ohwa Mental Clinic | Yoyogi Mental Clinic Institutional Review Board |
| Aiiku Clinic | Suzuki Internal & Circulatory Medical Clinic Institutional Review Board |
| Medical corporation Nobita Micri Kids Clinic | Mizuo Clinic Institutional Review Board |
| Kodomo Mental Clinic Shinotsuka | Mizuo Clinic Institutional Review Board |
| Tokai University School of Medicine | Tokai University Hospital Institutional Review Board |
| Nobisuko Children Clinic | Mizuo Clinic Institutional Review Board |
| Kishiro Mental Clinic | Mizuo Clinic Institutional Review Board |
| Sinsenkokoro-clinic | Dr. Mano Medical Clinic Institutional Review Board |
| Yokohama Onoecho Clinic | Yoyogi Mental Clinic Institutional Review Board |
| Tsuji Pediatrics Clinic | Fukui General Hospital Institutional Review Board |
| University of Fukui Hospital | University of Fukui Hospital Institutional Review Board |
| Hiratani Children’s Clinic | Fukui General Hospital Institutional Review Board |
| Shinshu University Hospital | Shinshu University Hospital Institutional Review Board |
| Azumino Internal Medicine Stress Care Clinic | Review Board of Human Rights and Ethics for Clinical Studies Institutional Review Board |
| Tenryu Hospital | Tenryu Hospital Institutional Review Board |
| HAMAMATSU City Child Psychiatric Clinic | Tenryu Hospital Institutional Review Board |
| Mikawa Hospital | Dr. Mano Medical Clinic Institutional Review Board |
| Child Clinic PaPa | Sugiura Clinic Institutional Review Board |
| Sugawara Clinic | Nishi Hospital Institutional Review Board |
| Osaka Asahi Children's Hospital | Sugiura Clinic Institutional Review Board |
| Nishimura Peditric Clinic | Nihonbashi Sakura Clinic　Institutional Review Board |
| Osaka Psychiatric Medical Center | Osaka Psychiatric Medical Center Institutional Review Board |
| Yasuhara Children’s Clinic | Nihonbashi Sakura Clinic　Institutional Review Board |
| Mikunigaoka Kokoro Hospital | Japan Conference of Clinical Research Institutional Review Board |
| Takahashi Psychiatric Clinic | Tokyo Allergy and Respiratory Disease Research Institute Clinical Trial Review Committee |
| Hibiki Mental Clinic | Kondo Memorial Medical Foundation Tomisaka Clinic Institutional Review Board |
| Nara Medical University Hospital | Nara Medical University Hospital Institutional Review Board |
| Kyo Mental Clinic | Dr. Mano Medical Clinic Institutional Review Board |
| Minami Wakayama Medical Center | Minami Wakayama Medical Center Institutional Review Board |
| Hiroshima-Nishi Medical Center | Hiroshima-Nishi Medical Center Institutional Review Board |
| Mukainada ekimae Mental Clinic | Dr. Mano Medical Clinic Institutional Review Board |
| Murakawa clinic | Sugiura Clinic Institutional Review Board |
| Matsuyama Municipal Hospital | Matsuyama Municipal Hospital Institutional Review Board |
| Ehime University Hospital | Ehime University Hospital Institutional Review Board |
| Fukuoka University Hospital | Fukuoka University Hospital Institutional Review Board |
| Kaku Mental Clinic | Sugiura Clinic Institutional Review Board |
| Parkside Kokoronohattatsu Clinic | Nihonbashi Sakura Clinic　Institutional Review Board |
| Mental Clinic Iris | Dr. Mano Medical Clinic Institutional Review Board |
| Saga Medical and Welfare Center for the challenged | Saga Memorial Hospital　Institutional Review Board |
| Sagaekiminami Clinic | Sugiura Clinic Institutional Review Board |
| Arata Clinic | Sugiura Clinic Institutional Review Board |
| Shimodoori　Psychosomatic medical clinic | Dr. Mano Medical Clinic Institutional Review Board |
| Miyakonojo Shinsei Hospital | Sugiura Clinic Institutional Review Board |
| Chikama Clinic | Sugiura Clinic Institutional Review Board |
| Kagoshima Prefecture Children's General Rehabilitation center | Dr. Mano Medical Clinic Institutional Review Board |

**Supplementary Table S3** Study device exposure and compliance

|  | Comparison part | Repetition part | |
| --- | --- | --- | --- |
|  | SDT-001  *N* = 109 | SDT-001/SDT-001 *N* = 75 | TAU/SDT-001 *N* = 51 |
| Device exposure (total number of days of the study device usage, days) | | | |
| Mean±SD | 35.3±7.3 | 35.0±8.6 | 34.6±8.3 |
| Median (min, max) | 37.0 (12, 45) | 38.0 (6, 45) | 37.0 (12, 43) |
| Proportion of use of the study device (%)^†^ | | | |
| Mean±SD | 84.1±16.1 | 83.8±18.0 | 82.3±18.8 |
| Median (min, max) | 90.2 (29.3, 100.0) | 89.7 (15.0, 100.0) | 89.5 (30.8, 100.0) |
| <50 | 6 (5.5) | 3 (4.0) | 5 (9.8) |
| ≥50 to <80 | 30 (27.5) | 17 (22.7) | 11 (21.6) |
| ≥80 | 73 (67.0) | 55 (73.3) | 35 (68.6) |

Data are mean±SD or *n* (%) unless specified otherwise.

^†^((number of days that completed 5 sessions) / (the latest date among the last date reported for status of use, visit 5, or discontinuation - the earliest date among the first date reported or visit 2 + 1)) × 100

max, maximum; min, minimum; SD, standard deviation; SDT-001, investigational digital therapeutic; TAU, treatment as usual.

**Supplementary Table S4** Change from baseline in ADHD-RS-IV (physicians’ assessment) at week 6 in comparison part

| Hierarchical order | Group | Change from baseline at week 6^†^ | | | | Cohen's d^‡^ |
| --- | --- | --- | --- | --- | --- | --- |
|  |  | n | Adjusted mean (Standard error) | Difference in adjusted mean [95% CI] | *P* value |  |
| #1 Primary endpoint: ADHD-RS-I (physician’s assessment) | TAU | 52 | -1.47 (0.65) |  |  |  |
|  | SDT-001 | 94 | -4.44 (0.49) | -2.97 [-4.38, -1.56] | <0.0001 | 0.65 |
| #2 Key secondary endpoint: ADHD-RS-T (physician’s assessment) | TAU | 52 | -2.46 (0.99) |  |  |  |
|  | SDT-001 | 94 | -7.02 (0.74) | -4.56 [-6.75, -2.28] | <0.0001 | 0.62 |
| #3 Key secondary endpoint: ADHD-RS-H (physician’s assessment) | TAU | 52 | -1.02 (0.49) |  |  |  |
|  | SDT-001 | 94 | -2.57 (0.37) | -1.55 [-2.64, -0.46] | 0.0056 | 0.39 |

^†^A mixed effect model for repeated measures with unstructured covariance was applied to the change from baseline in score from week 2 to week 6 as response. This model was based on group, timepoint (week 2 to week 6) and interaction between group and timepoint as fixed effects, value at the baseline, binarized age group (≤12 years, ≥13 years), prior ADHD medication (yes/no), and ADHD type (combined, predominantly inattentive, predominantly hyperactive-impulsive) as covariates. To control the family-wise Type I error rate, a fixed sequence, gatekeeping multiple testing procedure was applied to the primary endpoint and the key secondary endpoints. The primary endpoint was tested first at two-sided significance level of 0.05, and if statistically significant, then key secondary endpoints were tested in sequence.

^‡^Calculated as the arithmetic mean difference divided by a pooled standard deviation for the change from baseline.

ADHD, attention-deficit/hyperactivity disorder; ADHD-RS-H, attention-deficit/hyperactivity disorder rating scale IV - hyperactivity-impulsivity subscale scores; ADHD-RS-I, attention-deficit/hyperactivity disorder rating scale IV - inattention subscale scores; ADHD-RS-IV, attention-deficit/hyperactivity disorder rating scale IV; ADHD-RS-T, attention-deficit/hyperactivity disorder rating scale IV - total scores; CI, confidence interval; SDT-001, investigational digital therapeutic; TAU, treatment as usual.

**Supplementary Table S5** Summary of secondary endpoints at week 6 in comparison part

|  | SDT-001 *N* = 109 | | TAU *N* = 54 | | Comparison with TAU | |
| --- | --- | --- | --- | --- | --- | --- |
|  | *n* | Change from baseline  adjusted mean  [95% CI] | *n* | Change from baseline  adjusted mean  [95% CI] | Difference in  adjusted mean [95% CI] | *P* value |
| ADHD-RS-IV (teacher) inattentive subscale score^†^ | 96 | –0.05 [–1.00, 0.90] | 51 | 0.56 [–0.60, 1.72] | –0.61 [–1.79, 0.57] | 0.3106 |
| ADHD-RS-IV (teacher) total score^†^ | 96 | –0.7 [–2.4, 0.9] | 51 | 0.0 [–2.1, 2.0] | –0.7 [–2.8, 1.4] | 0.4984 |
| ADHD-RS-IV (teacher) hyperactivity-impulsivity subscale score^†^ | 96 | –0.7 [–1.6, 0.2] | 51 | –0.6 [–1.7, 0.5] | –0.1 [–1.2, 1.0] | 0.8554 |
| BRIEF monitor^†^ | 99 | –0.7 [–1.4, –0.1] | 53 | 0.3 [–0.5, 1.2] | –1.1 [–2.0, –0.2] | 0.0187 |
| BRIEF organization of materials^†^ | 99 | –0.3 [-0.8, 0.2] | 53 | -0.1 [-0.8, 0.6] | -0.2 [-0.9, 0.5] | 0.4946 |
| BRIEF plan/organize^†^ | 99 | –1.0 [–2.0, 0.0] | 53 | 0.0 [–1.3, 1.4] | –1.0 [–2.4, 0.3] | 0.1365 |
| BRIEF working memory^†^ | 99 | –1.0 [–1.9, –0.2] | 53 | 0.2 [–0.9, 1.3] | –1.2 [–2.4, –0.1] | 0.0339 |
| BRIEF initiate^†^ | 99 | –0.5 [–1.1, 0.1] | 53 | 0.2 [–0.6, 1.0] | –0.7 [–1.5, 0.1] | 0.0813 |
| BRIEF emotional control^†^ | 99 | –0.4 [–1.1, 0.3] | 53 | 0.4 [–0.5, 1.2] | –0.8 [–1.7, 0.1] | 0.0862 |
| BRIEF shift^†^ | 99 | –0.6 [–1.2, 0.0] | 53 | 0.2 [–0.6, 1.0] | –0.8 [–1.6, 0.0] | 0.0502 |
| BRIEF inhibit^†^ | 99 | –0.9 [–1.6, –0.2] | 53 | 0.1 [–0.8, 1.0] | –1.0 [–1.9, –0.1] | 0.0319 |
| BRIEF meta cognition^†^ | 99 | –3.6 [–6.6, –0.6] | 53 | 0.5 [–3.4, 4.4] | –4.2 [–8.1, –0.2] | 0.0385 |
| BRIEF behavioural regulation^†^ | 99 | –1.8 [–3.3, –0.2] | 53 | 0.6 [–1.4, 2.6] | –2.4 [–4.4, –0.4] | 0.0216 |
| Conners 3™ (parents) inattentive subscale score^†^ | 94 | –1.4 [–2.7, 0.0] | 52 | –0.3 [–1.9, 1.4] | –1.1 [–2.8, 0.6] | 0.2104 |
| Conners 3™ (parents) hyperactivity-impulsivity subscale score^†^ | 94 | –3.4 [–4.9, –1.8] | 52 | –2.0 [–3.9, 0.0] | –1.4 [–3.4, 0.6] | 0.1749 |
| Conners 3™ (parents) learning problems subscale score^†^ | 94 | –1.4 [–2.4, –0.4] | 52 | –0.9 [–2.1, 0.4] | –0.5 [–1.8, 0.7] | 0.4031 |
| Conners 3™ (parents) executive functioning subscale score^†^ | 94 | –0.1 [–1.0, 0.9] | 52 | 0.4 [–0.8, 1.6] | –0.5 [–1.7, 0.7] | 0.4078 |
| Conners 3™ (parents) aggression/defiance subscale score^†^ | 94 | –1.3 [–2.3, –0.2] | 52 | –0.4 [–1.7, 0.9] | –0.8 [–2.2, 0.5] | 0.2253 |
| Conners 3™ (parents) peer relations subscale score^†^ | 94 | –1.0 [–1.6, –0.3] | 52 | –0.5 [–1.3, 0.3] | –0.4 [–1.2, 0.4] | 0.3107 |
| Conners 3™ (parents) global index subscale score^†^ | 94 | –2.5 [–3.6, –1.3] | 52 | –1.7 [–3.1, –0.3] | –0.8 [–2.2, 0.7] | 0.3043 |
| Conners 3™ (parents) ADHD inattentive subscale score^†^ | 94 | –0.6 [–1.7, 0.6] | 52 | –0.2 [–1.6, 1.3] | –0.4 [–1.9, 1.1] | 0.5953 |
| Conners 3™ (parents) ADHD hyperactivity-impulsivity subscale score^†^ | 94 | –2.4 [–3.6, –1.1] | 52 | –1.5 [–3.1, 0.0] | –0.9 [–2.5, 0.8] | 0.2932 |
| Conners 3™ (parents) conduct disorder subscale score^†^ | 94 | –0.7 [–1.5, 0.1] | 52 | –0.3 [–1.3, 0.7] | –0.4 [–1.4, 0.7] | 0.4693 |
| Conners 3™ (parents) oppositional defiant disorder subscale score^†^ | 94 | –1.3 [–2.1, –0.5] | 52 | –0.7 [–1.7, 0.3] | –0.7 [–1.7, 0.4] | 0.2022 |
| Conners 3™ (parents) inattentive T score^†^ | 94 | –2.8 [–5.3, –0.2] | 52 | –0.4 [–3.6, 2.8] | –2.4 [–5.7, 0.9] | 0.1500 |
| Conners 3™ (parents) hyperactivity-impulsivity T score^†^ | 94 | –5.0 [–7.3, –2.7] | 52 | –2.5 [–5.4, 0.5] | –2.5 [–5.6, 0.5] | 0.1016 |
| Conners 3™ (parents) learning problems T score^†^ | 94 | –3.2 [–5.3, –1.1] | 52 | –1.7 [–4.3, 0.9] | –1.6 [–4.2, 1.1] | 0.2487 |
| Conners 3™ (parents) executive functioning T score^†^ | 94 | –0.4 [–2.4, 1.5] | 52 | 0.3 [–2.2, 2.8] | –0.7 [–3.2, 1.8] | 0.5735 |
| Conners 3™ (parents) aggression/defiance T score^†^ | 94 | –4.1 [–6.8, –1.3] | 52 | –1.5 [–4.9, 1.9] | –2.6 [–6.1, 1.0] | 0.1538 |
| Conners 3™ (parents) peer relations T score^†^ | 94 | –4.4 [–7.4, –1.5] | 52 | –1.2 [–4.9, 2.5] | –3.2 [–7.1, 0.6] | 0.0944 |
| Conners 3™ (parents) global index T score^†^ | 94 | –5.2 [–7.6, –2.7] | 52 | –3.2 [–6.3, –0.2] | –1.9 [–5.1, 1.2] | 0.2332 |
| Conners 3™ (parents) ADHD inattentive T score^†^ | 94 | –1.3 [–3.6, 1.0] | 52 | –0.7 [–3.5, 2.2] | –0.7 [–3.6, 2.3] | 0.6559 |
| Conners 3™ (parents) ADHD hyperactivity-impulsivity T score^†^ | 94 | –4.4 [–6.8, –2.0] | 52 | –2.6 [–5.7, 0.5] | –1.8 [–4.9, 1.4] | 0.2729 |
| Conners 3™ (parents) conduct disorder T score^†^ | 94 | –3.6 [–6.5, –0.7] | 52 | –2.4 [–6.0, 1.2] | –1.2 [–4.9, 2.5] | 0.5326 |
| Conners 3™ (parents) oppositional defiant disorder T score^†^ | 94 | –3.8 [–5.8, –1.8] | 52 | –2.0 [–4.5, 0.5] | –1.8 [–4.4, 0.8] | 0.1664 |
| IRS^‡^ | 94 | –0.72 (0.15) § | 52 | 0.09 (0.19) § | –0.81  [–1.24, –0.39] | 0.0002 |
| PedsQL™ generic core scales^‡^ | 99 | 3.45 (1.05) § | 53 | 1.15 (1.35) § | 2.29  [–0.56, 5.14] | 0.1147 |
| EQ-5D-Y (VAS)^‡^ | 94 | 2.55 (2.11) § | 52 | 4.21 (2.73) § | –1.66  [–7.48, 4.15] | 0.5724 |

^†^Analysis of covariance was performed on the change from baseline at week 6. This analysis was based on group as fixed effects, value at baseline, binarized age group (≤12 years, ≥13 years), presence of previous medication with indications for ADHD (yes/no), and ADHD type (combined presentation, predominantly inattentive presentation, predominantly hyperactive-impulsive presentation) as covariates.

^‡^Mixed effect model for repeated measures with unstructured covariance was applied to the change from baseline in score from week 2 to week 6 as response. This model was based on group, timepoint (week 2 to week 6) and interaction between group and timepoint as fixed effects, value at the baseline, binarized age group (≤12 years, ≥13 years), presence of previous medication with indications for ADHD (yes/no), and ADHD type (combined presentation, predominantly inattentive presentation, predominantly hyperactive-impulsive presentation) as covariates.

^§^Data presented as adjusted mean (standard error).

ADHD, attention-deficit/hyperactivity disorder; ADHD-RS-IV, attention-deficit/hyperactivity disorder rating scale IV; BRIEF, Behavior Rating Inventory of Executive Function; CI, confidence interval; EQ-5D-Y (VAS), EuroQol 5-Dimension Youth (visual analog scale); IRS, Impairment Rating Scale; PedsQL™, Pediatric Quality of Life Inventory; SDT-001, investigational digital therapeutic; TAU, treatment as usual.

**Supplementary Table S6** Proportion of participants with more than 30% improvement in ADHD-RS-IV (physician’s assessment) score at week 6 in comparison part

| ADHD-RS-IV (physician’s assessment) | SDT-001  *N* = 109 | | TAU  *N* = 54 | | Difference in proportion  [95% CI], *P* value^†^ |
| --- | --- | --- | --- | --- | --- |
|  | *n* | Responder  *n* (%) | *n* | Responder  *n* (%) |  |
| Total score | 94 | 33 (35.1) | 52 | 5 (9.6) | 25.3 [12.5, 38.1], 0.0010 |
| Inattentive subscale score | 94 | 27 (28.7) | 52 | 5 (9.6) | 18.8 [6.5, 31.1], 0.0092 |
| Hyperactivity-  impulsivity subscale score | 89 | 44 (49.4) | 52 | 7 (13.5) | 34.0 [19.6, 48.4], <0.0001 |

^†^Cochran-Mantel-Haenszel method stratified by age (≤12, ≥13 years), presence of previous medication with indications for ADHD (yes/no) and ADHD type (combined presentation, predominantly inattentive presentation, predominantly hyperactive-impulsive presentation).

ADHD, attention-deficit/hyperactivity disorder; ADHD-RS-IV, attention-deficit/hyperactivity disorder rating scale IV; CI, confidence interval; SDT-001, investigational digital therapeutic; TAU, treatment as usual.

**Supplementary Table S7** Change from baseline in ADHD-RS-IV (teacher’s assessment) scores in repetition part

| ADHD-RS-IV (teacher’s assessment) | Treatment  group | Comparison part  Baseline | | Comparison part  week 6  Change from baseline | | Repetition part  week 6  Change from baseline | |
| --- | --- | --- | --- | --- | --- | --- | --- |
|  |  | n | mean±SD | n | mean±SD | n | mean±SD |
| Inattentive subscale scores | SDT-001/SDT-001 | 75 | 13.8±6.7 | 70 | –0.8±3.9 | 68 | –2.5±4.2 |
|  | TAU/SDT-001 | 51 | 12.6±7.4 | 50 | 0.0±3.8 | 41 | –0.3±3.8 |
| Hyperactivity-impulsivity subscale scores | SDT-001/SDT-001 | 75 | 7.6±7.2 | 70 | –1.0±3.8 | 68 | –1.7±3.5 |
|  | TAU/SDT-001 | 51 | 8.8±7.4 | 50 | –0.8±3.2 | 41 | –0.6±3.7 |
| Total scores | SDT-001/SDT-001 | 75 | 21.4±12.6 | 70 | –1.8±6.8 | 68 | –4.2±6.9 |
|  | TAU/SDT-001 | 51 | 21.4±13.3 | 50 | –0.9±6.4 | 41 | –0.9±6.7 |

ADHD-RS-IV, attention-deficit/hyperactivity disorder rating scale IV; SD, standard deviation; SDT-001, investigational digital therapeutic; TAU, treatment as usual.
